# Model predicting social acceptance behavior to implement ELV policy: Exploring the role of knowledge toward ELV policy on social acceptance in Malaysia

**DOI:** 10.3389/fpubh.2022.1093732

**Published:** 2023-01-18

**Authors:** Hasani Mohd Ali, Charli Sitinjak, Muhamad Helmi Md Said, Jady Zaidi Hassim, Rozmi Ismail, Vladimir Simic

**Affiliations:** ^1^Faculty of Law, National University of Malaysia, Bangi, Malaysia; ^2^Centre for Research in Psychology and Human Well-Being, Faculty of Social Sciences and Humanities, National University of Malaysia, Bangi, Malaysia; ^3^Fakultas Psikologi, Universitas Esa Unggul, West Jakarta, Indonesia; ^4^Faculty of Transport and Traffic Engineering, University of Belgrade, Belgrade, Serbia

**Keywords:** end-of-life vehicles, contaminant, social acceptance, waste management, environmental sustainability, structural equating modeling

## Abstract

Effective management of end-of-life vehicles (ELVs) represents a sound strategy to mitigate global climate change. ELVs are contaminants that pollute water, air, soil, and landscape. This waste flow must be adequately treated, but no proper rule oversees the disposal of ELV waste in Malaysia. This study aims to determine the extent of implementing the ELV policy and the social readiness in implementing environmentally friendly ELV disposal in Malaysia. The questionnaire seeks public input on critical ELV concerns such as public perception of the phenomena, environmental and safety standards, and recycling and treatment facilities. This research uses a cross-sectional design with 448 respondents in the survey. Fit models in structural equation modeling are evaluated using a variety of goodness-of-fit indicators to ensure an actual hypothesis. This study's advantages include the availability of representative samples and allowing for comparable and generalizable conclusions to larger communities throughout Malaysia. It is found that personal experience is significantly correlated with social readiness. The cause of ELV vehicles knowledge was the vital mediator, along with recycling costs knowledge. Thus, knowledge regarding ELV management costs is the most decisive mediation variable to predict public acceptance. The recommended strategy to reduce resentment and rejection of ELV policy is to disseminate information about the negative ELV impact on environmental and social sustainability.

## 1. Introduction

Malaysia is one of the Southeast Asian nations with the most significant automobile sector. Malaysia has the third-highest automobile production rate in the Association of Southeast Asian Nations (ASEAN), behind Thailand and Indonesia, according to the Organization Internationale des Constructeurs d'Automobiles (OICA). Since the founding of PROTON in 1985 and PERODUA in 1993, the automobile sector in Malaysia has developed; this is a visible manifestation of the Malaysian government's national vehicle initiative (1).

The large production of vehicles in Malaysia positively affects the economy. However, the increasing mobility of automobiles utilized by Malaysians has led to urban air pollution, posing a severe threat to this warning (2–4). Even in 2019, the World Air Quality Index (WAQI) recorded a regional air pollution index of 299, making Malaysia the nation with the most significant amount of air pollution, followed by South Africa (217), Indonesia (207), India (194) and China (178) (5). This is a severe threat to Malaysian society, as the high levels of air pollution created by automobiles can lead to various health issues.

Air pollution is primarily caused by automobile emissions, especially in urban areas. Carbon monoxide (CO), nitrogen oxides (NOX), hydrocarbons, and sulfur oxides are produced mainly by automobile emissions (6–8). According to existing studies, carbon monoxide is the most prevalent contaminant, and over 70% of all carbon monoxide gases are generated by motor vehicles (9). Carbon monoxide is an odorless, tasteless, and non-irritating gas that has a lethal effect on humans despite its lack of color, odor, and taste (10–12).

Sulfur dioxide is an odorless gas with an offensive odor. The combustion of sulfur-containing fossil fuels produces sulfur oxide (13–15). This oxide contributes to acid rain and secondary particle production. Indirectly, the amount of sulfur emitted is proportional to the amount of sulfur in the fuel, primarily diesel (16–18). Automobile exhaust emissions also contain sulfur oxide, which is the primary source of sulfuric acid and the most prevalent form of acid deposition (19). Recent European research indicates that emission diesel contains sulfur in the form of particles and gases; hence reducing sulfur in diesel fuel will reduce particulates. Human exposure to dangerous substances is predicted to rise in densely populated metropolitan regions. This happens near busy traffic hubs in urban areas, where urban characteristics can lead to poor air dispersion, causing pollution hot spots (19–21).

Particulates (PM) and nitrogen dioxide (NO_2_) emitted from vehicle exhaust are often the primary sources of urban air pollution that negatively affect the health of people (22, 23). In 2016, on-road vehicles accounted for ~39 and 20% of the total NOx and CO emissions in the European Union (EU), and it was determined that passenger vehicles were the largest source of NOx and CO emissions from road transport (24). In addition to CO and total hydrocarbons (THC), diesel-powered vehicles have been recognized as the primary source of excessive NOx emissions in the EU (25–27).

Malaysia released 2,210,634 metric tons of carbon monoxide, 889,890 metric tons of nitrogen oxide, 257,457 metric tons of sulfur dioxide, and 26,789 metric tons of particulate matter in 2018 (28). PM10, which comes from power plants, accounts for 36% of the pollution; the remaining 63% comes from the industry; 16% comes from automobile exhaust; and 15% comes from residential, commercial, non-energy, and agricultural use (29). In 2018, 95.6% of Malaysia's carbon monoxide emissions came from automobiles (28).

Considering that the high number of automobiles has negatively impacted environmental and social sustainability, implementing the end-of-life vehicle (ELV) policy in Malaysia can be viewed as a solution for fostering the automotive industry's growth (30–32). As a nation that manufactures its automobiles, Malaysia is obligated to pay special attention to the impact of vehicles that contribute to the gradual contamination of the environment. The government made multiple attempts to formulate legislation to minimize car congestion on metropolitan highways in Malaysia, but they were discontinued due to widespread opposition.

ELV management and its recycling system are made up of several activities involving several processes. Effective and efficient ELV management is critical for maintaining the quality of the automotive industry's environment, economy, and ecosystem. As a result, ELV management has become a primary concern for authorities and researchers. In recent years, researchers have focused on reviewing relevant studies to understand better the dynamics of ELV management and the most appropriate recycling systems. Until now, most studies on ELV management and recycling systems have only focused on specific components such as ELV maintenance processes, demolition techniques, ELV management infrastructure, and ELV transportation facilities, but no one has focused on researching the social side to see the acceptance and public perception of ELV management.

For the first time, this study focuses on ELV review from a social standpoint as judged by personal experience, knowledge comprised of three factors (i.e., knowledge of the causes of ELV, knowledge of technological techniques, and recycling costs), as well as social readiness for implementing ELV. This study provides a comprehensive view of the Malaysian community's readiness for implementing the ELV policy. It has been discovered that knowledge can significantly increase the community's readiness to accept and implement ELV policies, even if their experience with ELV is still low.

The remainder of this paper is structured as follows. Section 2 provides a review of some related works. Section 3 presents the materials and methods. Section 4 shows the results. Section 5 gives a discussion of the results. Sections 6 and 7 provide managerial recommendations and research implications. Section 8 offers conclusions.

## 2. Literature review

### 2.1. Vehicles as a source of air pollution

Automobiles are the primary source of air pollution around the globe. The transportation industry is a significant contributor to traffic pollution. Besides, ELVs are eight times more likely to contribute to the increase in air pollution than new vehicles, and 15-year-old trucks contribute 10 times more to air pollution than new trucks (33, 34).

Large quantities of CO, THC, NOX, and hazardous chemicals such as fine particles and lead are emitted by motor vehicles (25–27). Private cars, motorcycles, light, and heavy vehicles all play a significant role in Malaysia's declining air quality, particularly in urban areas (33). These vehicles' emissions can negatively affect human health and the environment. This has led to the problem of severe air pollution accompanying the lives of urban residents (35, 36). Historically, air pollution concerns have been confined to metropolitan areas, but in recent years they have spread to impair the health of rivers, lakes, and forests (6, 19, 20). The impact of motor vehicle gases is beginning to have a highly negative influence on human health. This has led to concerns that motor vehicles have a catastrophic effect on global changes, which could alter the climate (37–39).

Sulfur oxides, CO, NOX, and THC are harmful gases to human health (40–42). CO is produced when fossil fuels are burned incompletely (43, 44). If humans inhale CO, they may have poisoning symptoms, including headaches, dizziness, nausea, vomiting, and loss of consciousness (27, 35, 45). CO was ligated to hemoglobin significantly more strongly than oxygen (33, 46). This demonstrates that persons exposed to CO to a certain degree frequently experience severe poisoning caused by the human body's loss of oxygen over a prolonged time. Additionally, CO is closely associated with global warming (47).

NOX is a traffic-related contaminant since automobile engines emit it (48, 49). As it penetrates deeply into the lungs, it irritates the respiratory system, causing respiratory illnesses, coughing, wheezing, dyspnea, bronchospasm, and even pulmonary edema when inhaled at high concentrations (50). It appears that concentrations >0.2 ppm cause these adverse effects in humans, whereas concentrations >2.0 ppm influence T-lymphocytes, specifically the CD8+ cells and NK cells that trigger our immune response (51). According to reports, prolonged exposure to high quantities of nitrogen dioxide can cause chronic lung illness.

Sulfur dioxide (SO_2_) is a toxic gas generated mainly by the combustion of fossil fuels and industrial processes. The annual SO_2_ standard is 0.03 ppm (50, 51). It affects humans, animals, and plants. Those susceptible, such as those with lung illness, the elderly, and youth, are more likely to sustain injury. The primary health issues associated with SO_2_ emissions in industrialized areas are respiratory irritation, bronchitis, mucus production, and bronchospasm because sulfur dioxide is a sensory irritant and penetrates deeply into the lung, where it is converted into bisulfite and interacts with sensory receptors, causing bronchoconstriction. In addition, redness of the skin, damage to the eyes (lacrimation and corneal opacity), and mucous membranes, as well as a worsening of cardiovascular disease, have been noted (52, 53).

In 2020, it was recorded that 31.2 million motor vehicles were registered in Malaysia, demonstrating that the automotive industry in Malaysia has multiplied. However, in the same year, the Road Transport Department reported that there are at least 60,000 inactive vehicles across the country whose registration expired more than 3 years, a matter of great concern (54). The development of the automotive industry can make Malaysia increasingly competitive with other developed countries in the automotive industry, stimulating economic growth in Malaysia. Nevertheless, there has been one problem behind the success: the more vehicles there are, the higher the risk of environmental pollution. According to 2019 statistics, CO produced by vehicles has been recorded as the worst pollution in Malaysia, accounting for 70.4% of the total pollution occurring. Abandoned vehicles that have not been used can also threaten the sustainability of the environment. This risk derives from waste materials such as electronic components, lithium batteries, lead acid batteries, used engine oil, engine coolant, etc. When left without proper management, these waste materials will result in indirect environmental pollution (33).

The Public Complaints Bureau received numerous complaints from 2014 to 2017 regarding abandoned vehicles in Malaysia, where 15,019 cases of abandoned vehicles were totaled for the year (54). However, there has been an increase in the number in 2019, when the Road Transport Department informed that there are at least 60,000 abandoned vehicles across the country (54). The facts and statistics on abandoned vehicles are highly concerning since they not only affect environmental degradation but can also impact the welfare of the country's community; i.e., social sustainability. Many abandoned automobiles are parked in public places such as roadsides, parking lots, and other areas where they can annoy the public. Vehicles parked on the side of the road contribute to visual pollution. This pollution is an aesthetic issue that can interfere with a person's ability to enjoy a sight or vista (55). Destructive natural changes have disrupted an individual's visual area, resulting in visual pollution. As a result, the abandoned vehicle might produce visual pollution in the surrounding population, resulting in distraction, eye fatigue, a reduction in the diversity of opinions, and loss of identity.

### 2.2. ELV policy in Malaysia

The ELV policy is a source of contention in Malaysia. Many people rejected this policy but the Malaysian Institute of Road Safety Research, which highlighted possible hazards in passenger vehicles older than 12 years, gradually implemented it. There were almost four million cars in Malaysia older than 10 years old in 2017, which is expected to rise to 9.97 million by 2020. According to the research of Azmi and Tokai (56), the rapid growth of ELVs in Malaysia will lead to new problems with environmental pollution. Moreover, an ELV policy can contribute to long-term environmental control while also boosting safety in more modern vehicles (57). Furthermore, if Malaysia adopts the ELV policy, the country's economy will expand and the new car assessment program for Southeast Asian Countries will be more successful (1).

As one nation that produces automobiles, the Malaysian government has not yet established an ELV legislation. This is evidence that there is no desire from the automobile industry sector to implement the ELV policy, even though the total industry volume has reached 500,000 vehicles during the past decade (1).

Malaysians have been interested in ELV policy since 2009 (30). The Ministry of International Trade and Industry had evaluated this regulation progressively at the time, even though it was not included in the initial policy. This policy had several essential elements, including annual car inspections for 15 years. However, the public quickly criticized this regulation, so it was quickly abandoned. The ELV policy was then recreated in 2014 (30). However, it was not explicitly specified, and the word was substituted with Voluntary Vehicle Inspection (VVI). The Malaysian government's intention when introducing VVI was to reduce carbon intensity in metropolitan Malaysia by 40%.

In 2014, the National Automotive Policy (NAP) also began emphasizing the roadworthiness of automobiles to guarantee that they were safe to operate. As part of formulating future ELV laws, NAP introduced the Automotive Remanufacturing Roadmap and the Authorized Treatment Facilities Roadmap (57).

In 2016, the Malaysian government also launched the “cash-for-clunkers” initiative, which gave a cash rebate system to reduce the number of ELVs on the road (58). However, the rejection came not only from the community but also from the minister of transportation, who delivered a reaction that was unsupportive of giving instructions that every regulation must be further considered before implementation because it can have detrimental effects on several parties. Additionally, the 4R program was launched in 2016 (59). This program offers “Reuse, Recycle, Remanufacture, and Repair Service and Spare Parts” and has attracted the participation of 34 Malaysian enterprises. The objective of the 4R program in Malaysia is to promote the automobile sector and discourage the use of older vehicles (60).

Malaysia launched a new automotive policy in February 2020 (61). This policy emphasizes the growth of the automotive manufacturing sector, engineering, and technology. However, precise directions for ELV management are no longer discussed. Local automotive ambitions to transform Malaysia into an automotive manufacturing hub in ASEAN have resulted in the abandonment of ELV-related negotiations and a singular concentration on production and market expansion. According to Karagoz et al. (62), the management of ELV can only be successful if a single government enacts ELV-related policies. Besides, ELV management must incorporate the entire ecosystem, from the last vehicle owners to related centers and facilities for dismantling, recycling, and remanufacturing.

Malaysia may take lessons from advanced jurisdictions in regulating ELVs. For example, the European Parliament approved Directive 2000/53/EC on ELVs (i.e., ELV Directive). As a result, all EU member states adopted it in regulating the environmentally friendly ELV management process that prioritizes the reuse, recycling, and recovery of ELVs (63).

Germany is among the member states of the EU that have successfully adopted the Directive into their legal system. As a result, the recycling rate of ELVs in the country reaches 87.7%, exceeding the reuse and recycling target set by the ELV Directive (64).

Apart from Germany, Ireland can also be an excellent example in the disposal and management system of ELVs. The recycling rate and recovery of ELVs in Ireland show an 87.43% recycling rate and a 95.21% recovery rate in 2019. It is estimated that 160,000 cars are disposed of each year in Ireland, most of which are between 10 and 16 years old (65). Any last vehicle owner will receive a certain amount of payment when disposing of an ELV. However, insufficient disposal fees were paid in the late 1990's and early 2000's, leading to an increase in abandoned vehicles (65). However, according to Farrell (66), experts in vehicle disposal predicted that in 2022, the value of an ELV would rise. The increase can be seen from November and December 2021, when the value of an ELV increased significantly to more than €320 compared to the average ELV value in 2020, which is as much as €100. This increase is very significant (66). It can encourage car users and owners to dispose of their ELVs responsibly. Additionally, ELV must dispose of and processed only at locations that have been registered following Irish government regulations.

Raja Mamat et al. (31), surveyed the factors that can lead to success in establishing an end-of-life vehicle management system in Malaysia without considering the performance level of local ELV management. The subsequent study conducted by Abu Kassim et al. (1) analyzed only the public's response to the probability of implementing the ELV management policy. However, it did not include any information on the current practice of ELV management in Malaysia. Therefore, it can be outlined that no study has been done on the readiness of the Malaysian public to accept an ELV policy.

### 2.3. End-of-life vehicles process

There are two different kinds of ELV: natural and premature (67). Premature vehicles have reached the end of their useful life sooner than expected. This could be because of a fire, theft, flood, destruction, or damage from an accident. In this case, often a part can be used again before going on to the next step. Natural ELV, on the other hand, is a vehicle that has reached the end of its useful life (59–68).

When taking apart an ELV, the first step is to take out the batteries, liquids, and other potentially dangerous parts. Then, high-value parts are dismantled, and parts that still work are sold directly to secondary markets. The remaining parts of the vehicle are then forwarded to recyclers and processors. After removing the iron, the non-ferrous scrap can be separated using the solid medium separation process (69, 70).

The ELV processing is split into two parts: disassembly and destruction during the process and recycling and energy recovery after the process (71, 72). Parts with high economic value are picked out in traditional ways in a typical disassembly process. These parts are resold or fixed up. On the secondary market, usable parts can be sold directly to users (71, 73).

The evolving manufacturing technique may pose a certain issue. For example, the ELV Directive requires a 95% recovery rate from ELV and an 85% recycling rate. However, recent changes in vehicle material composition indicate a gradual replacement of ferrous metals traditionally used in the automotive industry with plastics and composites, to reduce vehicle weight, fuel consumption, and CO_2_ emissions. As a result, the share of recyclable materials decreases while the share of materials that are difficult (and thus expensive) to recycle increases (74).

For the shredding process, the body of the car and any broken parts will be sent to a crushing plant, where they will be broken down into smaller pieces that are easier to handle. The ELV will be pressed down before it goes into the shredder. The leftover pieces of the car, called automobile shredder residue (ASR), are then separated by type. Magnets are used to separate metals from other types of materials (75, 76).

### 2.4. Knowledge predicting social acceptance

Knowledge is a significant factor in a person's acceptance. Knowledge factors are deemed capable of explaining an individual's acceptance and can functionally contribute to greater acceptance by enhancing an individual's understanding of the rules and their intended purpose. The more an individual's awareness about regulations, the greater the likelihood they will comply. In such circumstances, understanding the rules positively affects a person's ability to receive without a response. According to Sitinjak et al. (67), a high environmental regulation knowledge correlates with solid support for their implementation. Similarly, Modoi and Mihai (77), demonstrated that the objective level of information could positively influence social and economic attitudes, as well as acceptance of ELV policy. On the other hand, according to Baldassarre et al. (78), the higher the public's awareness of the impact of ELV on the environment, the greater the positive influence on society's acceptance of ELV regulation.

In the instance of air pollution, knowledge might indirectly affect individuals' acceptance of efforts to safeguard the surrounding environment by highlighting the risks and advantages of successfully implementing the rules. Marvi et al. (79) revealed that a person's acceptance of environmental regulations was heavily influenced by social perceptions, environmental attitudes, and personal experiences. Furthermore, it was discovered that environmental knowledge positively correlates with acceptance, especially when viewed from the perspective of education level (80, 81). Education has a more significant effect on people's desire to protect and maintain their environment. Conversely, the less education a person has, the more difficult it is to convince them to accept the norm (78).

## 3. Materials and methods

The research was conducted in several parts of Malaysia, such as Pahang, Selangor, Melaka, Perlis, Pulau Pinang, Johor, Kedah, Negeri Sembilan, Terengganu, Perak, Kelantan, Sabah, Sarawak, and the Guild Region. The collection of areas is used for data gathering since there are many cases of old vehicles remaining in operation and even left unattended on the periphery of the road and public parking lots without suitable treatment.

### 3.1. Study design

This research uses a cross-sectional design. The cross-sectional method is used by researchers because all variables are measured and observed at the same time in this study design, making it easier for researchers to conduct research and obtain relevant results (82). Krejcie and Morgan (83), suggested that the minimum required total sample size was 384 if the population was up to 1 million. In this research, 448 respondents took part in the survey. This survey selected respondents who were 18 or older and in possession of a valid driver's license using a systematic random sampling method. In this study, all enumerators and trained personnel explained the goal of the survey to selected respondents. Only respondents who provided written authorization were permitted access to the research. Respondents' socio-demographic backgrounds (age, gender, race, education, employment, income, vehicle ownership, etc.), knowledge (ELV causes, recycling techniques, recycling costs), and readiness were collected in their houses. The survey sheets filled out by respondents are then examined by using SPSS and WAR-PLS. The human research committee of Universiti Kebangsaan Malaysia has approved this study (UKM PPI/111/8/JEP-2021-595).

### 3.2. Study instrument

**Figure 1:**
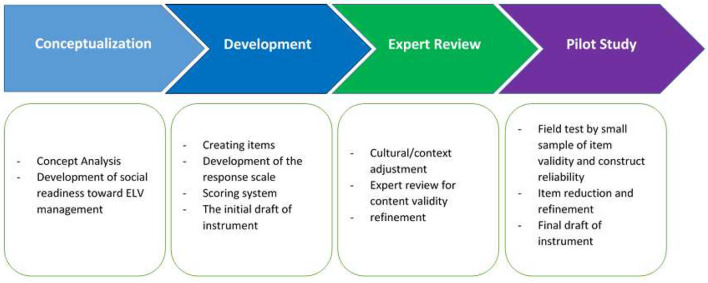
Instrument development and validation procedure.

To address the issues raised in this study, the Trans-Disciplinary Research Grant Scheme (TRGS) team from the Faculty of Law developed a set of questions based on previous literature and interviews with numerous experts (see Figure 1 for instrument development and validation steps). We planned several question sessions for this investigation. The first session was used to collect respondents' demographic information, the second session was used to gain respondents' experiences with the vehicles they owned, and the third session was used to prepare questions about respondents' knowledge of ELV management (ELV causes, recycling techniques, recycling costs), and the final session was used to determine respondents' readiness to accept ELV. Cronbach's alpha for the total item is more than 0.05, which is excellent. The values for the scales of experience, understanding of ELV causes, recycling processes, and recycling prices are 0.83, 0.87, 0.79, and 0.82, respectively, and 0.87 for the scale of community readiness.

### 3.3. Criteria for model evaluation

Fit models in structural equation modeling (SEM) are evaluated using a variety of goodness-of-fit indicators. Because chi-squared tests are susceptible to sample size, many descriptive models of fit indexes have been created over time. A double match index was used in this work to examine the model fit between hypothetical models and sample data. In addition, some match indexes are taken into account when compared to the basic model. Approximate benchmarks for good model fit are values of 0.95 or higher for the Comparative Fit Index (CFI) and Tucker-Lewis Index (TLI) and values of 0.08 or less for Root Mean Square Error Approximation (RMSEA) and Standardized Root Mean Residual (SRMR). Suppose the null hypothesis of no difference is not rejected at each stage of the invariant test. This implies that the parameter limitation does not result in a worse solution than the base model, and the invariant hypothesis is empirically confirmed.

## 4. Results

The characteristics of the survey respondents are shown in Table 1. A total of 448 responses were received from 14 Malaysian states, with men accounting for 54.02% (*n* = 242) and women accounting for 45.98% (*n* = 206). The age range of respondents in this survey ranged from 26 to 35 years old (31.70%) to 56 years and older (7.81%). The bulk of respondents in this survey was Malay (72.10%), followed by respondents with an undergraduate degree education level of 34.82% (*n* = 156), and there were as many as 0.67% (*n* = 3) respondents who did not attend formal institutions. Respondents in this study also owned vehicles (*n* = 369), which was in agreement with the researcher's goals, as researchers would examine the experiences of respondents who had vehicles with their readiness to adopt the requirements of the ELV policy.

**Table 1:** Sample characteristics and key frequencies.

**Total *N* = 448**					
**Gender**	**Total**	**%**	**State of residence**	**Total**	**%**
Male	242	54.02	Pahang	6	1.34
Female	206	45.98	Selangor	79	17.63
	448		Melaka	12	2.68
**Age**			Perlis	34	7.59
25 years and under	82	18.30	Pulau Pinang	4	0.89
26–35 years old	142	31.70	Johor	22	4.91
36–45 years old	139	31.03	Kedah	5	1.12
46–55 years old	50	11.16	Negeri Sembilan	6	1.34
56 years and older	35	7.81	Terengganu	2	0.45
	448		Perak	90	20.09
**Race**	**Total**	**%**	Kelantan	43	9.60
Malay	323	72.10	Sabah	34	7.59
Chinese	41	9.15	Sarawak	82	18.30
Indian	26	5.80	Wilayah Persekutuan	29	6.47
Bumiputera Sabah	19	4.24		448	
**Bumiputera Sarawak**	39	8.71	**Occupation**	**Total**	**%**
	448		Private sector	109	24.33
Educational level	**Total**	**%**	Government sector	226	50.45
No formal education	3	0.67	Self-employed	46	10.27
Primary school	4	0.89	Student	38	8.48
Secondary school	102	22.77	Unemployed	22	4.91
College/ STPM/ diploma	122	27.23	Retiree	7	1.56
Undergraduate degree	156	34.82		448	
**Postgraduate masters**	52	11.61	**Vehicles ownership**	**Total**	**%**
Doctor of philosophy	9	2.01	Yes	369	82.37
	448		No	79	17.63

### 4.1. Validity

The validity test results show the loadings value of each indicator and cross-loading to determine the validity of the instruments used in the study. The table shows the results of validity testing where it can be seen that the load factor > 0.5 and *p* < 0.001, then the variables tested in this study meet the convergent validity (see Table 2).

**Table 2:** Combination of loadings and cross-loadings.

	***Z*1**	***Z*2**	***Z*3**	***X***	***Y***	**Type**	**SE**	***P*-value**
*Z*1-1	0.763	0.018	0.057	0.000	0.074	Reflective	0.043	<0.001
*Z*1-2	0.730	0.010	−0.068	−0.011	−0.100	Reflective	0.043	<0.001
*Z*1-3	0.785	−0.007	0.027	−0.017	0.070	Reflective	0.043	<0.001
*Z*1-4	0.666	−0.024	−0.022	0.032	−0.057	Reflective	0.043	<0.001
*Z*2-1	0.005	0.804	−0.037	−0.031	0.062	Reflective	0.043	<0.001
*Z*2-2	0.072	0.801	−0.051	0.025	0.031	Reflective	0.043	<0.001
*Z*2-3	0.037	0.744	0.047	−0.019	−0.116	Reflective	0.043	<0.001
*Z*2-4	0.047	0.733	0.049	0.026	0.016	Reflective	0.043	<0.001
*Z*3-1	0.052	0.111	0.695	−0.027	0.063	Reflective	0.043	<0.001
Z3-2	0.009	0.165	0.709	0.008	0.054	Reflective	0.043	<0.001
Z3-3	−0.061	0.130	0.722	−0.012	−0.015	Reflective	0.043	<0.001
Z3-4	−0.037	−0.177	0.649	0.013	−0.057	Reflective	0.043	<0.001
Z3-5	0.035	−0.026	0.731	0.017	−0.047	Reflective	0.043	<0.001
X-1	−0.116	0.124	−0.157	0.614	0.268	Reflective	0.044	<0.001
X-2	−0.053	0.010	−0.015	0.774	0.049	Reflective	0.043	<0.001
X-3	−0.100	0.056	0.028	0.762	0.041	Reflective	0.043	<0.001
X-4	−0.120	0.110	−0.097	0.657	−0.051	Reflective	0.043	<0.001
X-5	0.149	−0.102	0.048	0.721	−0.099	Reflective	0.043	<0.001
X-6	0.108	−0.035	0.024	0.660	−0.042	Reflective	0.043	<0.001
X-7	0.124	−0.141	0.139	0.722	−0.141	Reflective	0.043	<0.001
Y-1	0.058	0.048	0.058	0.104	0.770	Reflective	0.043	<0.001
Y-2	0.057	−0.076	0.070	0.005	0.858	Reflective	0.042	<0.001
Y-3	0.047	−0.087	0.076	0.029	0.803	Reflective	0.043	<0.001
Y-4	−0.033	−0.012	−0.039	−0.038	0.833	Reflective	0.042	<0.001
Y-5	−0.058	0.113	−0.091	−0.045	0.809	Reflective	0.043	<0.001
Y-6	−0.036	0.078	−0.122	−0.065	0.822	Reflective	0.043	<0.001
Y-7	−0.035	−0.060	0.051	0.017	0.805	Reflective	0.043	<0.001

X, Personal experience; Z1, Knowledge ELV causes; Z2, Knowledge recycling techniques; Z3, Knowledge recycling cost; Y, Social readiness.

Table 3 illustrates that the AVE root on the major diagonal has a bigger value than the correlation to the variable under consideration. The variable *Z*_1_ AVE root value of 0.737, for example, is bigger than 0.272, 0.061, 0.151, and 0.392. These findings indicate that the variable *Z*_1_ satisfies the discriminant's validity. Similarly, the root value of the AVE is bigger than the correlation to other factors for other variables.

**Table 3:** AVE root and correlation coefficient.

	***Z*1**	***Z*2**	***Z*3**	***X***	***Y***
*Z*1	0.737	0.272	0.061	0.151	0.392
*Z*2	0.272	0.771	0.516	0.322	0.598
*Z*3	0.061	0.516	0.702	0.321	0.497
*X*	0.151	0.322	0.321	0.703	0.295
*Y*	0.392	0.598	0.497	0.295	0.815

X, Personal experience; Z1, Knowledge ELV causes; Z2, Knowledge recycling techniques; Z3, Knowledge recycling cost; Y, Social readiness.

### 4.2. Reliability

**Figure 2:**
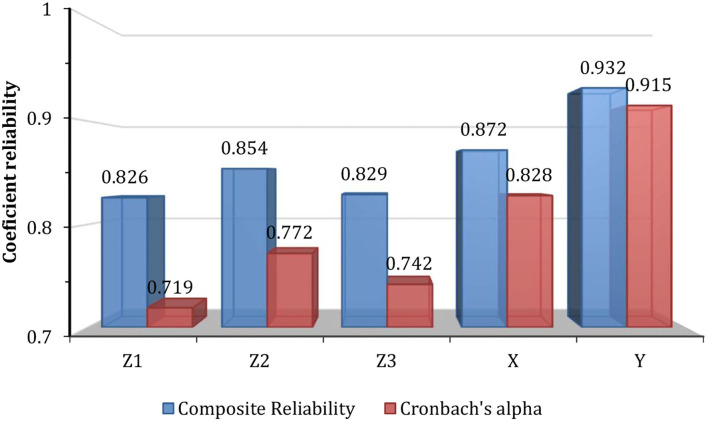
Composite reliability and Cronbach's alpha values. *X*, Personal experience; *Z*1, Knowledge ELV causes; *Z*2, Knowledge recycling techniques; *Z*3, Knowledge recycling cost; *Y*, Social readiness.

Furthermore, the composite reliability value is used to determine the reliability test. If the composite reliability coefficient exceeds 0.7, the measuring instrument is declared to meet the composite reliability. Figure 2 presents the composite reliability coefficient values of all > 0.7 and Cronbach's alpha > 0.5, indicating that it meets the reliability standard.

### 4.3. Descriptive and bivariate analysis

We categorize respondents' responses into three groups: opponents, neutrals, and supporters. Bivariate analysis is utilized to illustrate the significant variations between each element. The majority of respondents rejected the ELV policy, as demonstrated by these results. Here from the results of the analysis, it was also determined that household income has a significant influence on a person's acceptance or rejection of the ELV policy (*p*-value 0.043) and that the duration of stay has the most significant influence on a person's acceptance of the ELV policy (*p*-value 0.005) (see Table 4).

**Table 4:** Comparison between respondents supporting and opposing ELV management.

**Demography factors**	**Opponents**	**Neutral**	**Supporters**	***P*-value**	
Gender	Male	229 (98%)	4 (2%)	-	0.493
	Female	211 (98%)	4 (2%)	-	
Education	No formal education	1 (100%)	-	-	0.598
	Primary school	4 (100%)	-	-	
	Secondary school	104 (100%)	-	-	
	College/ STPM/ diploma	109 (97%)	3 (3%)	-	
	Undergraduate degree	154 (97%)	5 (3%)	-	
	Postgraduate masters	58 (100%)	-	-	
	Doctor of philosophy	10 (100%)	-	-	
Age	25 years and under	73 (97%)	2 (3%)	-	0.321
	26–35 years old	141 (99%)	1 (1%)	-	
	36–45 years old	139 (99%)	2 (1%)	-	
	46–55 years old	60 (99%)	1 (1%)	-	
	56 years and older	27 (93%)	2 (7%)	-	
Household income	Below RM2,500	138 (99%)	1 (1%)	-	0.043
	RM2,501–RM5,000	124 (99%)	2 (1%)	-	
	RM5,001–RM8,000	78 (99%)	1 (1%)	-	
	RM8,001–RM11,000	56 (99%)	1 (1%)	-	
	RM11,000 and above	44 (94%)	3 (6%)	-	
Tenure of stay	< 1 year	32 (100%)	-	-	0.005
	1–10 years	214 (99%)	1 (1%)	-	
	11–20 years	100 (99%)	1 (1%)	-	
	21–30 years	63 (94%)	4 (6%)	-	
	31–40 years	20 (91%)	2 (9%)	-	
	41 years and above	11 (100%)	-	-	
Vehicle ownership	Yes	366 (99%)	5 (1%)	-	0.124
	No	74 (99%)	3 (1%)	-	

### 4.4. General measurement model

To test the relationship between the direct and indirect variables of ELV management acceptance designed by the researchers in this study, several variables have been chosen to create an acceptance model based on individual experience, knowledge as a mediator, and readiness as the final output. We employ the SEM technique to answer our research questions. We also utilized this method to determine how our model influenced Malaysians' adoption of the ELV policy. The WarPLS 8.0 application aids in the analysis of research data.

**Figure 3:**
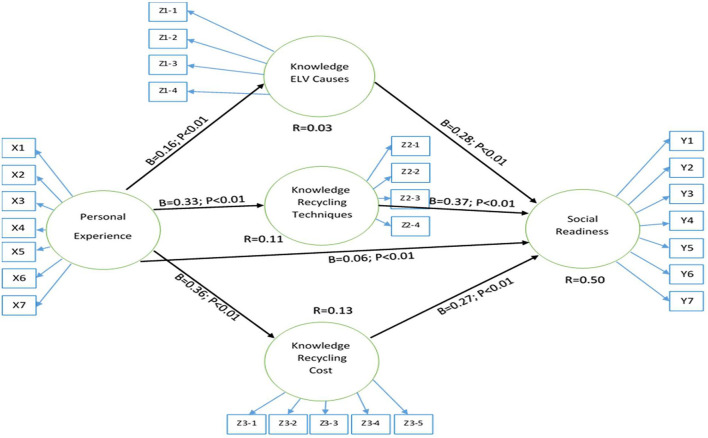
The final path model with standardized regression coefficients.

Figure 3 illustrates the relationship between the variables that influence one another. Personal experience was found to be significantly correlated with social readiness with indigo = 0.06; *P* 0.001, and the knowledge related to the cause of ELV vehicles were found to be the mediator that strengthened the relationship between personal experience and social readiness, with a total effect of total = 0.44; *P* 0.001. With a discernible influence of = 0.63 and a significance level of *P* 0.001, knowledge regarding recycling costs was also found to be a good mediator. Thus, it can be concluded that knowledge regarding ELV management costs is the strongest mediation variable that can predict public acceptance. Finally, it was discovered that knowledge regarding recycling techniques could not be a good mediator because its significance level is more significant than 0.05 (*P* = 0.100). Overall, the regression model has an excellent fit; all outcomes satisfy the fir model's requirements and quality indexes (see Table 5). Therefore, it can be stated that the constructed model adequately describes social acceptability.

**Table 5:** Model fit and quality indices result.

**Model fit and quality indices**	**Cut of value**	**Analysis result**	**Model**
Average path coefficient (APC)	Accepted if *p* < 0.005	0.001	Fit
Average R-squared (ARS)	Accepted if *p* < 0.005	0.001	Fit
Average adjusted R-squared	Accepted if *p* < 0.005	0.001	Fit
Average blok VIF (AVIF)	Accepted if ≤ 5	1.292	Fit
Average full collinearity VIF	Accepted if ≤ 5	1.534	Fit
Tenenhaus GoF (GoF)	Small > 0.1 Medium > 0.25 Large > 0.36	0.38	Medium
Simpson's paradox ratio	Accepted if ≥ 0.7	1	Fit
R squared contribution ratio	Accepted if ≥ 0.9	1	Fit
Statistical supprenssion ratio	Accepted if ≥ 0.7	1	Fit
Non-linear bivariate causality direction ratio (NLBCDR)	Accepted if ≥ 0.7	1	Fit

## 5. Discussion

Increasing ELV management knowledge, ELV management costs, and individual experience affect readiness, as shown by the findings of this study. A lack of awareness regarding ELV has fueled the public's opposition to implementing an ELV policy in Malaysia. This study has therefore explained why the government must increase its knowledge regarding the causes of ELV and the expense of maintaining ELV-containing vehicles, as knowledge has been shown to have a significant impact in this study.

This study's findings are consistent with prior research conducted in Indonesia, which indicates that a society's acceptance or rejection of ELV management norms can be predicted primarily by its level of knowledge (67). Other studies have demonstrated that an individual's acceptance of the norms governing ELV management is influenced by his or her experience and understanding of the effects and dangers posed by ELV (84–86). Government measures, such as increasing negative effect campaigns from ELV vehicles, must continue to be developed and presented to the public as one explanation. Many government efforts, including the ELV management program, have not directly impacted various social circles (87, 88). The availability of platforms for continually disseminating information is crucial to facilitate information access for the entire community.

In this study, those who disagreed with and rejected ELV management policies imposed in Malaysia had a very high presentation, which showed that people's willingness to accept ELV's activities and knowledge are linked positively. This fits with research that shows rejection is higher when people do not know much about something (89). In the same way, researchers in Indonesia found that insufficient individual knowledge is linked to rejection (90). Concerning knowledge and rejection, a study about the barriers to implementing ELV management in developing countries shows that one of the biggest reasons for rejection is that the government does not educate the public about the policies they will implement (4).

Several factors, such as environmental knowledge upgrading programs, can be implemented at the individual, community, and societal levels to elevate social values (91, 92). Social media can increase knowledge at the individual level by delivering broadcasts or adverts directly to each user. Community-level interventions are implemented to enable communities in more prominent groups to develop a shared environmental consciousness (93). For instance, environmental awareness programs that the government can implement in homes or villages to foster a sense of social trust can serve as a conduit for public acceptance of ELV regulations. In addition, its campaigns in broadcast media, the internet, etc., can be a readily accessible way of expanding public awareness. This demonstrates the need to enhance public awareness so that the community is more prepared and willing to adopt the ELV laws advocated by this study.

## 6. Managerial recommendations

According to the rapidly increasing number of ELVs in Malaysia, where no specific regulation in dealing with ELV exists, the government and researchers continue to seek the best solution to promote and implement ELV policies in Malaysia. Therefore, this research aims to ascertain how well-received the proposed ELV policy implementation will be in Malaysia. The study's findings show that the general public still needs to gain a greater understanding of ELV policies, mainly due to a lack of information about ELV vehicles, their management, and the potential harm they can cause. People's sentiment toward recycling their old cars is primarily influenced by the memories they have made behind the wheel.

Based on the findings, this study suggests different ways for the government to make rules about EVL that are acceptable to the public. Most importantly, this study shows that the government should work to improve the public's knowledge of ELV management, especially about why vehicles become ELVs, how to recycle them, and the costs of maintaining ELVs that will be paid for by owners who still want to use them. The government must also develop good ways to educate the public, such as through ads, flyers, ELV-related campaigns, and learning about the benefits of implementing ELV management. The government also needs to be able to fix problems with how people think about ELV recycling to help and encourage people to use a standard, environmentally friendly way to recycle ELV.

In addition, it was discovered that there are still a limited number of operators in Malaysia who can manage ELV following the established standards. Therefore, the Malaysian government must construct an ELV-related integrated management platform. In addition, the government must comprehend the costs associated with the ELV recycling process and the benefits realized if the ELV policy is successfully implemented so that economic barriers, which are one of the social barriers, can be eliminated. Malaysia should also advance recycling and reproduction technologies for ELV waste management.

## 7. Research implications

- In terms of limitations, this study tried to develop a model of community readiness for ELV management policies. However, in its development, researchers should have paid more attention to the acceptance model related to policies that had previously been made. Therefore, it is crucial to consider the policy acceptance model for future studies.
- In addition, with the development of the automotive sector in Malaysia, researchers must also explore in depth the competition between used car dealers and new car dealers to get richer results in seeing the problems that occur from an industry perspective.
- In this study, the offered acceptance model has limitations related to factors that describe community acceptance/readiness in the implementation of ELV, so we advise researchers to expand by adding various factors that can predict social acceptance of policies to get more comprehensive results in looking at community acceptance.
- We recommend employing a variety of research methodologies to delve deeper; e.g., qualitative methods or the use of mixed methods to produce more relevant results in the future.

## 8. Conclusions

Environmental sustainability is the ultimate aim of ELV policy. The results of this study provide numerous stakeholders with the information they need to establish suitable intervention programs to raise the understanding of all Malaysians, as has been done in several nations that have effectively implemented ELV-related legislation. Overall, the findings of this study indicate the need to increase public knowledge about ELV management, particularly at the community level, among all stakeholders, to achieve a solid understanding of the causes of ELV, the cost of ELV treatment, and the effects of ELV on health and the environment. According to the findings of this study, the public is more concerned about the cost of ELV treatment than the effects on health and the environment. In this study, it is also possible to demonstrate to stakeholders that the high level of community rejection is due to the absence of community-specific regulations. The results of the study also indicate that, even though individuals have positive recollections of their automobiles, they are more likely to comply with ELV management regulations if they are aware of the harmful effects of ELVs on environmental and social sustainability. Some techniques, such as advertising and ELV-targeted campaigns, can provide possibilities for widespread knowledge expansion. In addition, community members can organize meetings to discuss ELV-related concerns.

This study's advantages include the availability of representative samples, allowing for comparable and generalizable conclusions to larger communities throughout Malaysia. The report also contains extensive information regarding Malaysian society. Nonetheless, this study has limitations that must be overcome. This included the absence of ethnic additions in this study, as well as the absence of any consideration of naming criteria connected to prior ELV treatment. Moreover, to obtain better findings, future research could include other elements that predict acceptance, such as attitude, trust, etc. the restrictions of the discoverable are also flaws of this study.

## Data availability statement

The original contributions presented in the study are included in the article/supplementary material, further inquiries can be directed to the corresponding author.

## Author contributions

HA and CS: conceptualization, investigation, writing—review and editing, supervision, project administration, and funding acquisition. MM and JH: conceptualization, project administration, review, and editing. RI, CS, and VS: finalization. All authors have read and agreed to the published version of the manuscript.
